# Clinical Outcome of Free Vascularized Fibula Graft for Nonunion of Garden IV Femoral Neck Fracture of 13 Years Duration: A Case Report

**DOI:** 10.1111/os.13154

**Published:** 2022-03-29

**Authors:** Yangming Zhang, Yongguang Liu, Qulun Yan, Zhou Xiang

**Affiliations:** ^1^ Department of Orthopedics West China Hospital, Sichuan University Chengdu China; ^2^ Department of Orthopedics People's Fourth Hospital of Sichuan Province Chengdu China

**Keywords:** Free vascularized fibula graft, Nonunion, Old femoral neck fractures, Young patients

## Abstract

**Background:**

Femoral neck fractures in young patients are mostly caused by high‐energy trauma and demonstrate more displacement and vertical fracture surfaces, which increase nonunion and osteonecrosis risks. Free vascularized fibula graft (FVFG) is effective in treating old femoral neck fractures and nonunion; however, available data are limited to patients within 2 years after injury or revision surgery. We present the case of a patient who was diagnosed with femoral neck fracture at the age 9 and treated with FVFG 13 years later.

**Case presentation:**

A 9‐year‐old Asian girl who experienced left hip pain after an injury was diagnosed with Garden IV left femoral neck fracture, which was treated through manipulation reduction and fixed with splints. At age 16, the pain worsened after another injury and was considered to be in the physical development stage. She refused surgical treatment; hence, the fracture was fixed externally with splints. At age 22, she was hospitalized owing to a 12‐day left hip pain with restricted movement caused by a fall. She was diagnosed with old Garden IV femoral neck fracture nonunion and treated with FVFG. Seven years postoperatively, imaging showed that the left femoral neck was internally fixed, the fracture had healed, and the Harris score was 90 points. The 36‐Item Short Form Health Survey responses revealed that the patient's physiological functioning, emotional well‐being, energy, and mental health were normal. She achieved satisfactory functional results and resumed her normal daily life.

**Conclusion:**

FVFG could provide satisfactory outcomes for long‐term old femoral neck fractures.

## Graphical abstract

The patient had 13 years history of femoral neck fracture. After FVFG operation, the patient was able to participate in daily work normally.

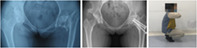

## Introduction

Femoral neck fractures often occur in elderly patients with osteoporosis. Due to poor local blood flow, the incidence of fracture nonunion and femoral head necrosis is higher in the elderly than in younger patients. In these patients, artificial joint replacement is the primary therapy^1^. For patients <50 years, the demand for hip joint preservation has led to the development of various surgical procedures. The commonly used surgical procedures include osteotomy, non‐vascularized bone graft, muscular pedicle bone graft, and vascularized bone graft^2^, ^3^, ^4^, ^5^, ^6^. Free vascularized fibula graft (FVFG) has been used to treat juvenile femoral neck fracture nonunion after surgery, or femoral neck fractures within 2 years of injury. However, there are few reports on the use of FVFG as the first operation in young patients with long‐term old femoral neck fractures. In this study, we described the application and outcome of FVFG in a 13‐year nonunion femoral neck fracture in a young adult, and this report highlights the satisfactory outcome.

## Case Presentation

**Fig. 1:**
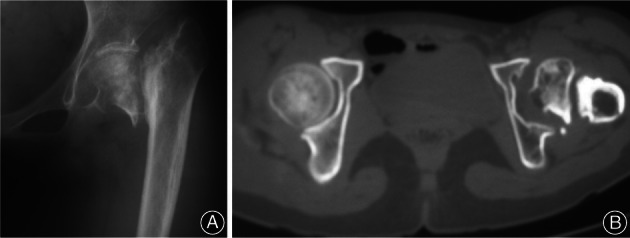
Radiographs and computed tomography images of femoral neck at the patient 16 years old shows nonunion of Garden IV femoral neck fracture.

In January 2000, a 9‐year‐old Asian girl presented with left hip pain following an injury, and was diagnosed with Garden IV left femoral neck fracture, which was treated by manipulation reduction and fixed with splints (no imaging examination). Although the pain improved, the left lower limb (LLL) became shortened by approximately 1 cm. In May 2006, the pain in the left hip worsened, following another injury at the age of 16 years. She was then diagnosed with evolving old Garden IV femoral neck fracture (Fig. 1). The patient refused surgical treatment, and was treated with external splints, which were removed after 1 month, and the pain was relieved. The visual analogue scale score was 3 points. The LLL was shortened by approximately 2 cm with claudication. In May 2013, at 22 years old, the patient was hospitalized with left hip pain following a fall, with limited mobility for 12 days. On physical examination, the LLL was lame, the left hip was limited in motion, passive hip flexion was about 60°, abduction and adduction were limited, movement of the artificial joint was evident, the left hip was more significantly atrophied than the right, the LLL was shortened by about 3.5–4.0 cm, muscle strength in the LLL was 3–4, while sensation and reflexes in both lower limbs were normal.

**Fig. 2:**
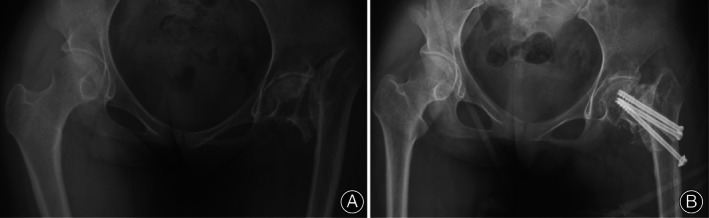
Radiographs of the patient's pelvis during this admission reveal a Garden IV femoral neck, old fractures are not union (A) and Postoperative review of the pelvis show left femoral neck fracture is well reduced and the fibula graft is in good position (B).

On admission, imaging showed (Fig. 2A) Garden IV left femoral neck old fracture with nonunion, and that the left femoral trochanter had moved up by 1.5 cm, the femoral neck stump was hardened, the medullary cavity was closed, and scattered free bone fragments were seen around the joint. Before surgery, her Harris score was 25, and a tibial traction of 8 kg was applied to loosen the soft tissues to provide space for restoring the length of the LLL, and to facilitate intraoperative reduction. After 3 weeks of traction, the surgery was performed.

### Surgical Technique

The technique described in a previously published paper^7^ was modified for this patient. Briefly, a longitudinal incision, approximately 9.0 cm long, was made on the left calf along the fibula, nearing two‐thirds its length. Subsequently, the left fibula was dissected and cut to about 6.0 cm, with the vascular pedicle of the fragment being approximately 2.0 cm. A 10‐cm longitudinal incision was made under the anterior superior iliac spine, anterior to the left hip joint. The articular capsule was cut to expose the left femoral neck, fibrous tissue, and hypertrophic scar tissue; and the nonunion site was removed. Transaction and reduction of the LLL were performed, and the neck shaft angle was increased to improve weight‐bearing. The remaining part of femoral neck was shortened by approximately 2 cm to obtain maximum contact area of the broken end, to prevent nonunion. The autologous fibula with its blood vessels was inserted into the bone groove which was made on the anterosuperior part of the left femoral neck, along the direction of the femoral neck. The distal end of the fibula was fixed internally using an absorbable screw. Three hollow compression screws were implanted under the greater trochanter of the femur for internal fixation in a regular triangular shape. The deep branch of the left lateral femoral circumflex artery was anastomosed with the peroneal artery and its accompanying veins under a microscope, and the distal vessel was ligated, with improved blood flow.

The patient lost approximately 1500 mL of blood and received 4 U of red cell suspension. The operation lasted for approximately 6 h and was successful.

### Postoperative Rehabilitation

On postoperative day 1, the patient was administered low‐molecular‐weight heparin and antibiotics to prevent infection, and 8 kg of tibia bone traction was used to maintain the force line. On postoperative day 3, she was instructed to perform passive exercises for lower limb function and muscle strength. On postoperative day 15, the bone traction was removed and replaced with herringbone plaster external fixation. Crutches were used for movement without weight‐bearing, and the plaster was removed after 2 months. Three months after surgery, the patient was able to walk and returned to her normal life.

### Clinical Outcome

**Fig. 3:**
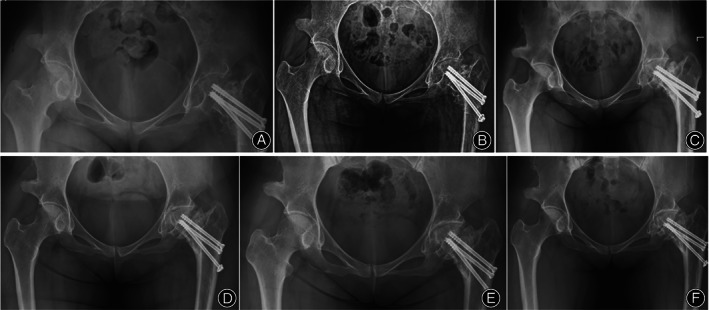
patient's orthopedic pelvic radiographs 1 month (A), 3 months (B), 1 year (C), 3 years (D), 5 years (E), 7 years (F) after surgery.

**Fig. 4:**
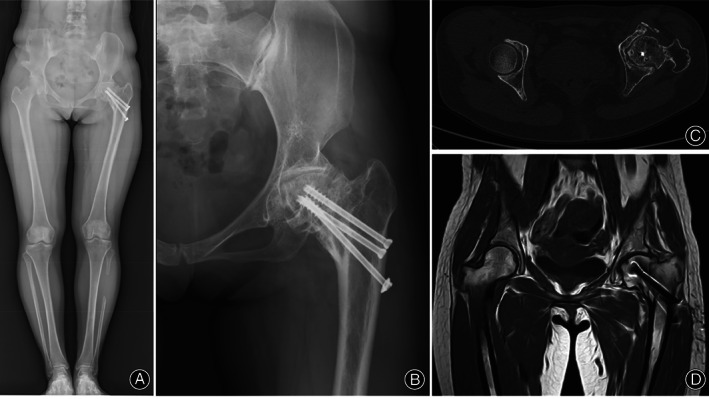
The total length of the lower limbs of the patient (A), X‐ray (B), CT (C), and MRI (D) of the patient's femoral neck 7 years after surgery.

**Fig. 5:**
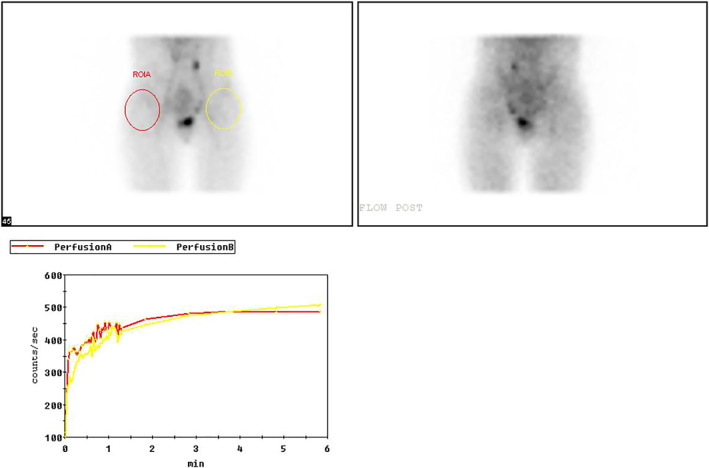
Bone scan showed that the patient's blood perfusion and blood volume of the hip joint area on both sides were not abnormal 7 years after surgery.

Routine follow‐up imaging (Fig. 2B) showed that the left femoral neck fracture was well reduced and that the fibula graft was in good position. The left femoral neck was 2 cm shorter than the right one, and the neck shaft angle was approximately 115°. The surgical incisions healed completely, and stage1healing was observed. Imaging examinations were performed at 1 month (Fig. 3A), 3 months (Fig. 3B), 1 year (Fig. 3C), and 3 (Fig. 3D) and 7 (Fig. 3F) years after surgery. One month after the operation, an X‐ray showed stable internal fixation of the femoral neck and good placement of the implanted bone. Imaging at 3 months after the operation showed healing of the femoral neck fracture and visible continuous callus formation. The femoral neck fracture healed completely and the implanted bones were fused at 1 year after surgery. Seven years after the surgery (Fig. 4), imaging examination revealed the following: the left femoral neck was internally fixed and stable, the femoral neck fracture was completely healed, the left femoral neck was about 2 cm short, the left hip joint showed bone hyperplasia, and the implanted bones had completely fused. Magnetic resonance imaging (Fig. 4D) revealed uneven signal from the left femoral head, with a small cystic low‐density shadow. Bone scan demonstrated (Fig. 5) no definite bilateral abnormalities with blood perfusion and blood volume in the hip joint area.

**Fig. 6:**
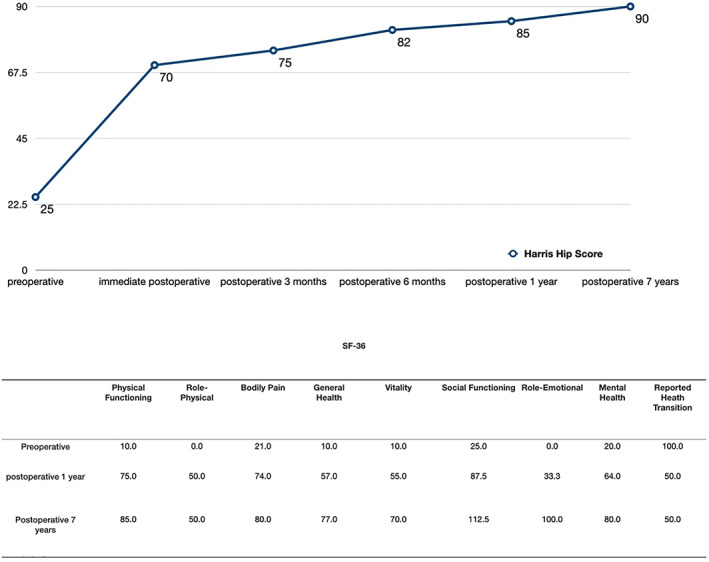
Harris hip joint function scores and SF‐36 scores of the patient during the treatment and follow‐up period.

**Fig. 7:**
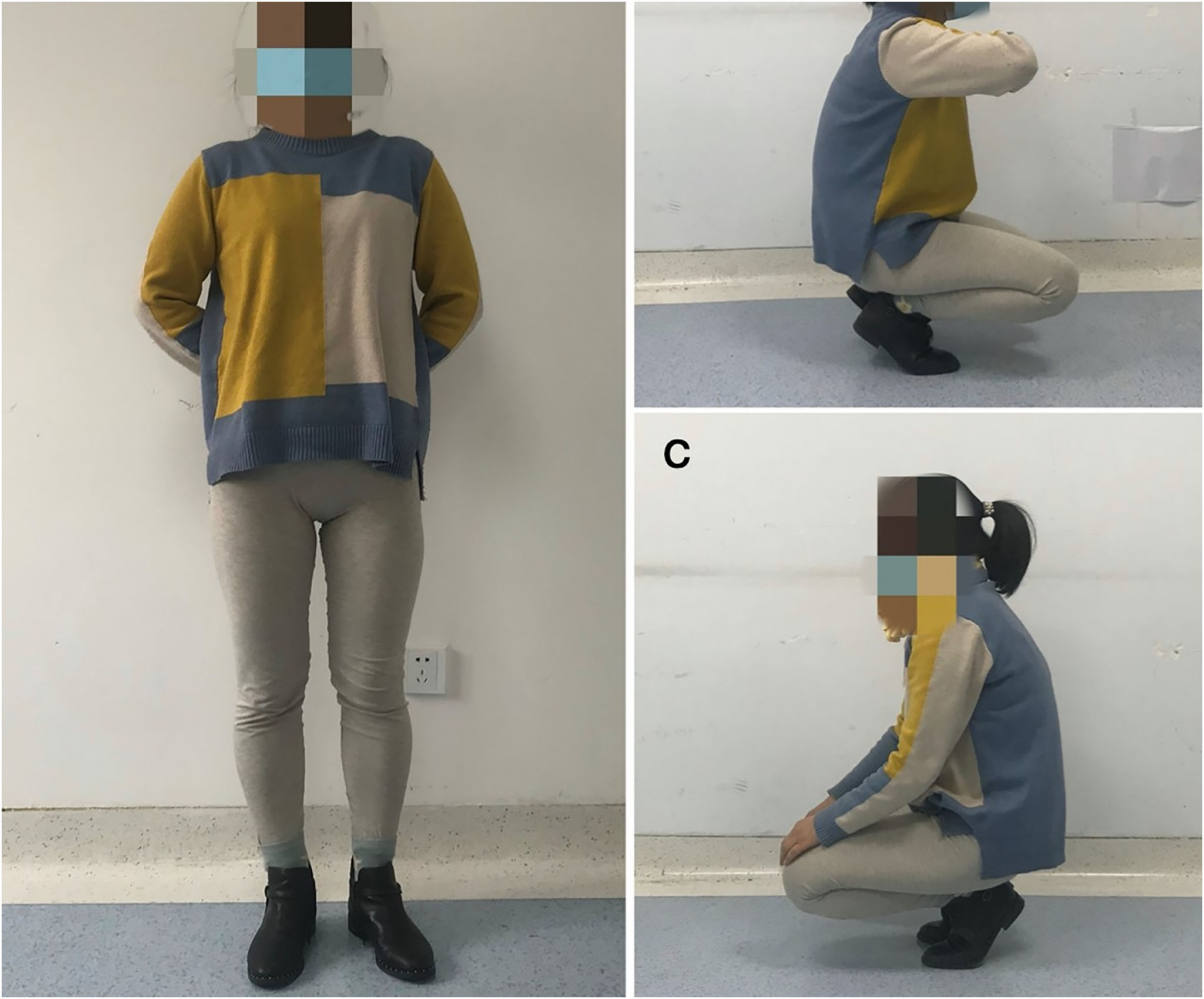
The patient's general function 7 years after surgery.

The Harris score for left hip joint function was evaluated at 3 months, 6 months, 1 year, and 7 years postoperatively. The Harris hip joint function scores gradually increased to 75, 82, 85, and 90 (Fig. 6). SF‐36 scores were obtained before surgery and at 1 year and 7 years after the operation (Fig. 6). The last follow‐up was 7 years after the surgery, when the patient was 29 years old. She had no obvious claudication during walking. The hip joint of the LLL had active flexion of about 105°, passive flexion of about 115°, abduction of 35°, adduction of 30°, and normal muscle strength. Gait analysis showed that her walking speed, gait cycle, and stride length were all normal. She could walk and climb stairs without assistance (Fig. 7) and had resumed normal work.

## Discussion

The incidence of nonunion of femoral neck fractures and avascular necrosis of the femoral head in young people is approximately 25%–90%^8^. Artificial joint replacement is mainly used in elderly patients, whereas the treatment of young patients is more inclined toward retaining the femoral head.

As early as 1975, Taylor *et al*. used FVFG to repair bone defects in the extremities^9^, whereas Judet *et al*. reported the first successful treatment of early femoral head necrosis with this surgical technique in 1988^10^. In recent years, a large number of studies^11^, ^12^, ^13^, ^14^, ^15^ have confirmed that FVFG can not only heal femoral neck bone nonunion, but also significantly reduce the rate of femoral head necrosis. Vascular fibular grafting is better than simple internal fixation and is especially suitable for young and middle‐aged patients with femoral neck fractures.

FVFG can simulate the general fracture healing process. For bone defects in weight‐bearing parts such as the femoral neck, a large number of cancellous bone implants cannot replace the original weight‐bearing strength, and dense bone implants that do not anastomose blood vessels increase the healing process. In addition to providing a new blood supply for the fractured end and providing a better condition for vascular repair, the fibula implanted in the femoral neck fracture site can prevent trabecular bone fractures and collapse of the femoral head. Moreover, it can play a favorable role in the internal fixation of fractures. As a dense bone, fibula implantation can stimulate the proliferation of bone cells in the bone graft area for a long time and promote blood supply reconstruction and tissue repair in femoral head necrosis.^16^, ^17^, ^18^, ^19^, ^20^, ^21^, ^22^.

Reports on the repair mechanism and clinical effects of FVFG have proven that this is a reliable treatment method for young patients with femoral neck fractures. However, the reported cases were restricted to injuries <2 years, or cases that required revision surgery after failure of the first operation. In theory, the longer the fracture time of the femoral neck fracture, the greater the possibility of femoral head necrosis. In this case, the operation will be more difficult, the probability of fracture healing will be reduced, and the function of the hip joint after surgery is not ideal. However, our patient achieved excellent functional outcome, with no signs of femoral head necrosis. We speculate that this was attributed to the increased neck shaft angle which changed the line of force and reduced the vertical shear force. Furthermore, considering the patient's economic situation, we chose to loosen the soft tissue through tibial traction before surgery. Preoperative traction and early postoperative functional exercises contributed to this good clinical outcome.

### Conclusion

In our case, FVFG could help achieve satisfactory clinical outcome even with 13 years nonunion of Garden IV femoral neck fracture that developed through adolescence. This result may broaden the surgical indication of FVFG, but needs additional original clinical studies for confirmation.

## Ethics Approval and Consent to Participate

This study was conducted in accordance with approval from the Ethics Committee of People's Fourth Hospital of Sichuan Province (Chengdu, China). Written informed consent has been provided by the patient. And the patient gave us full permission for the materials to appear in the print and online, and grant permission to third parties to reproduce this material.
